# Cell free HIV-1 virus can infect inner and outer foreskin polarized explants

**DOI:** 10.1186/1742-4690-9-S2-O22

**Published:** 2012-09-13

**Authors:** MP Lemos, ML Perez, J Sanchez, J Lama, S Montano, J McElrath

**Affiliations:** 1 Fred Hutchinson Cancer Research Center, Seattle, WA, USA; 2 IMPACTA, Lima, Peru; 3 Naval Medical Research Unit 6, Lima, Peru

## Background

In sexually insertive men, HIV is predominantly transmitted at the penile surface. We report two mayor advances in the study of HIV infections at the foreskin.

## Methods

First, we have developed cryopreservation methods that allow infection of foreskin tissue explants after thawing (654 +/- 178.3 pg of p24/gr of tissue) and provide comparable infection rates to fresh samples( 716.5 +/- 446pg of p24/gr of tissue). Second, we have developed an ex vivo assay that uses human foreskin explants to replicate the polarized viral entry. After 12h of polarized exposure, HIV entering the explant is amplified for 6 days of culture with activated PBMCs and measured using p24 ELISA.

## Results

Preserving the epithelial barriers, we show that polarized infections permit the entry and expansion of less virus (68.28 +/- 10.65pg of p24/gr of tissue) than non-polarized infections (650.4 +/- 205.9pg of p24/gr of tissue) where the virus can enter the CD4 T cell rich epidermal-dermal interface (p=0.04). Using 10000 TCID50 of HIV-1Bal per explants, we can detect infection in 100% of the non-polarized assays and 69% of the polarized explants. Lastly, comparing foreskin tissue from 4 donors, we demonstrate that the inner and outer foreskin are both equally able to support cell-free HIV infection in polarized assays (inner 77.67 +/- 64.7 vs. outer 92.82 +/- 66.81pg p24/gr of tissue p=0.369) and in non-polarized assays (inner 730.9 +/- 323.7 vs. outer 625 +/ 301.2pg p24/gr of tissue p=0.41).

## Conclusion

We hope that this approach could be used efficiently as a model to evaluate the efficacy of prevention strategies.

